# Association of statin use and left ventricular function in patients with breast cancer receiving trastuzumab

**DOI:** 10.1016/j.ahjo.2026.100871

**Published:** 2026-09-05

**Authors:** Kotaro Nochioka, Yosuke Terui, Aika Udagawa, Shintaro Kato, Shigeru Toyoda, Masaya Shimojima, Yasuhiro Izumiya, Toshiyuki Yano, Takeshi Kitai, Satoshi Yasuda, Koichiro Sugimura

**Affiliations:** a Department of Cardiovascular Medicine, Tohoku University Graduate School of Medicine, Sendai, Japan; b NEC Solution Innovators, Ltd, Japan; c Department of Cardiovascular Medicine, Dokkyo Medical University, Tokyo, Japan; d Department of Cardiovascular Medicine, Graduate School of Medical Sciences, Kanazawa University, Kanazawa, Japan; e Department of Cardiovascular Medicine, Graduate School of Medical Sciences, Kumamoto University, Kumamoto, Japan; f Department of Cardiovascular, Renal and Metabolic Medicine, Sapporo Medical University School of Medicine, Sapporo, Japan; g Department of Heart Failure and Transplantation, National Cerebral and Cardiovascular Center, Osaka, Japan; h Department of Cardiology, International University of Health and Welfare Narita Hospital, Narita, Japan

## Abstract

**Aims:**

We examined the association between statin use and changes in left ventricular ejection fraction (LVEF) during trastuzumab therapy in women with breast cancer. Methods: This post-hoc analysis of the CHECK HEART-BC study included 215 patients with normal baseline LVEF and 12-month echocardiographic follow-up. We assessed LVEF at baseline and at 3, 6, 9, and 12 months by statin use.

**Results:**

Of 215 women, 31 (14%) were receiving statins. Baseline LVEF was comparable between groups. After a similar decline at 3 months, LVEF trajectories diverged, with better preservation among statin users apparent at 9 months and most pronounced at 12 months. Statin use was associated with attenuated LVEF decline over time in an adjusted longitudinal model (interaction estimate, 0.27; standard error, 0.10; *P* = 0.005).

**Conclusions:**

Statin use was associated with better LVEF preservation during trastuzumab therapy, particularly during later follow-up. These findings are hypothesis generating and require confirmation in randomized trials.

## 1 Introduction

Breast cancer is the most common cancer among women, and chemotherapy that includes a Human epidermal growth factor receptor 2 (HER2) inhibitor, trastuzumab, improves prognosis, whereas it can also cause cardiac dysfunction [1]. Recent basic and clinical data suggest that statins may have cardioprotective effects against both anthracycline- and trastuzumab-associated cardiotoxicity [2], [3], [4], [5], [6], [7], [8]. However, much of the available clinical evidence, including meta-analyses [6] and randomized trials [7], [8], has focused primarily on anthracycline-related cardiotoxicity in patients with breast cancer or lymphoma. Because anthracycline-induced myocardial injury and HER2 inhibition-related cardiac dysfunction involve distinct mechanisms, evidence derived from anthracycline-treated populations cannot be directly extrapolated to trastuzumab-associated cardiotoxicity. Therefore, the potential cardioprotective effect of statins during trastuzumab therapy remains incompletely understood. To investigate the association between statin use and longitudinal changes in left ventricular ejection fraction (LVEF), we conducted a post-hoc analysis of a multicenter prospective observational study (Comprehensive HEart imaging to evaluate Cardiac damage linKed with cHEmotherApy in bReasT cancer patients; CHECK HEART-BC) [9] in female patients with breast cancer receiving trastuzumab with or without anthracyclines.

## 2 Methods

Female patients with breast cancer scheduled for neoadjuvant or adjuvant chemotherapy between August 2017 and March 2020 were prospectively enrolled (*n* = 643). Clinical data were collected using an electronic database system. Cardio-oncology surveillance—including echocardiography, electrocardiography, and biomarker evaluation—was performed every 3 months for 12 months following initiation of chemotherapy. Echocardiography was performed at each site by certified sonographers according to American Society of Echocardiography guidelines. LVEF was measured using the biplane Simpson method with manual endocardial tracing and automated calculation at each site. Baseline continuous and categorical variables were analyzed using *t*-tests and chi-square tests, respectively. Longitudinal changes in LVEF were evaluated using an adjusted longitudinal model including age, hypertension, ACEI/ARB use, anthracycline, baseline LVEF, and statin use. The study complied with the Declaration of Helsinki and was approved by the Ethics Committee of Tohoku University (ID: 2018–1-132). All patients provided written informed consent.

## 3 Results

Among 643 women enrolled in CHECK HEART-BC (mean age 56 ± 12 years), we sequentially excluded 19 patients with inappropriate timing of chemotherapy initiation, 333 patients who did not receive trastuzumab, 2 patients with a baseline LVEF ≤53%, and 74 patients with missing LVEF measurements at one or more visits. A total of 215 female patients receiving trastuzumab were included in the final analysis (**Supplemental Fig. 1 and Supplemental Table 1**). Of these, 31 (14%) were receiving statins at baseline and 184 (86%) were not. Among the 31 patients receiving statins at baseline, 30 (96.8%) had a history of dyslipidemia. Of these 31 patients, 23 (74.2%) continued statin therapy throughout the 12-month follow-up period. Among the 184 patients who were not receiving statins at baseline, 7 (3.8%) initiated statin therapy during follow-up.

**Fig. 1:**
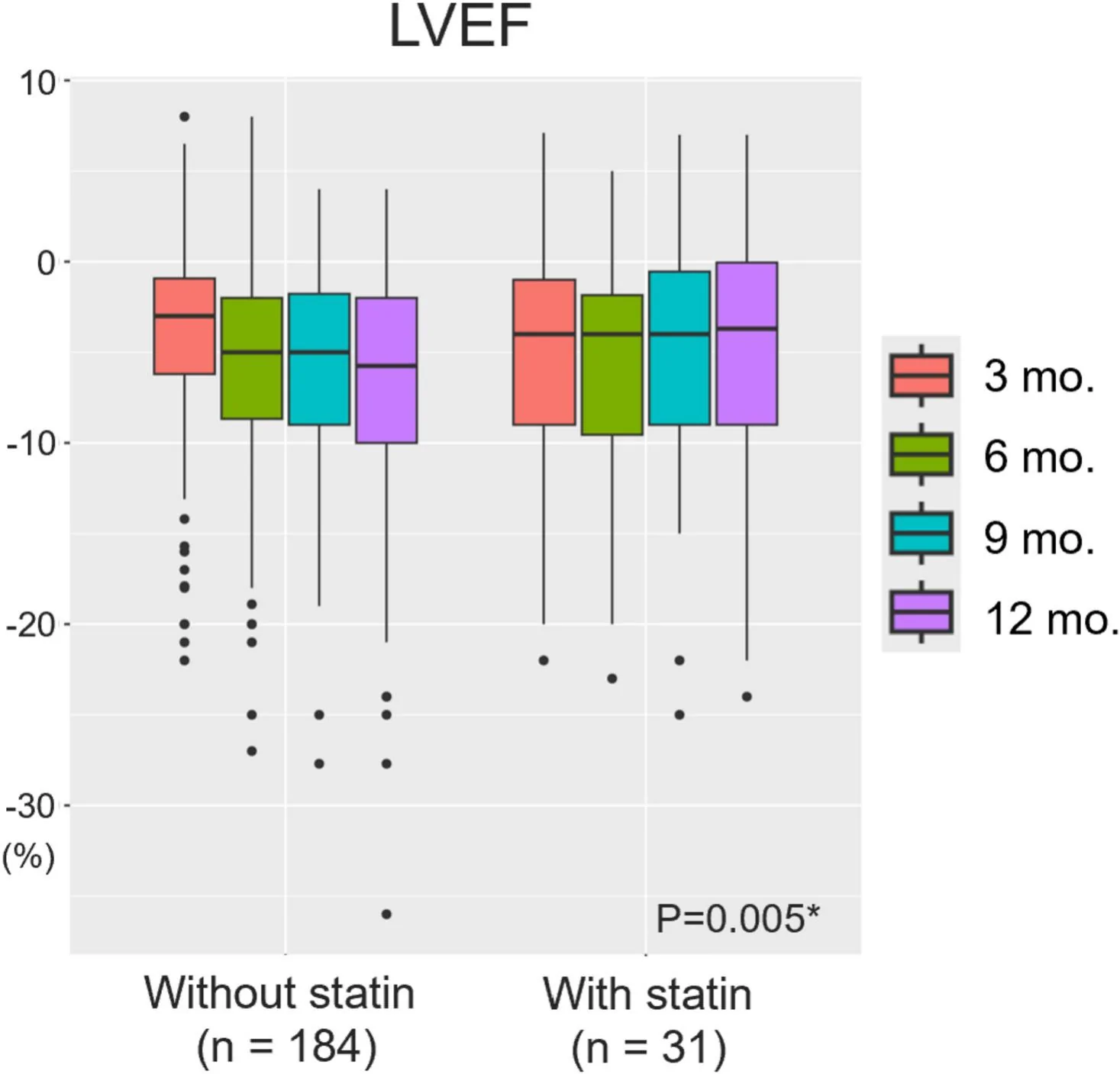
Longitudinal changes in left ventricular ejection fraction (LVEF) from chemotherapy initiation to 12 months stratified by statin use (184 without statin and 31 with statin; complete-case analysis). *Adjusted for age, hypertension, ACE inhibitor/angiotensin receptor blocker (ACEI/ARB) use, anthracycline exposure, and baseline LVEF in the longitudinal model.

HER2 positivity was confirmed by biopsy in 100% and 97% of the statin group and non-statin group, respectively (*p* = 0.80). Cancer staging was similar between two groups (Stage I/II; 32%/52% of the statin and 29%/39% of the non-statin groups). The mean age of the overall cohort was 57 ± 11 years, with statin group being significantly older than non-statin group (65 ± 10 vs. 55 ± 11 years, *p* < 0.001). The statin group had a higher prevalence of cardiovascular risk factors: Hypertension was observed in 61% in the statin group compared with 19% in the non-statin group, diabetes mellitus in 16% vs. 7%, and dyslipidemia in 97% vs. 17% (all *p* < 0.001). The statin group had a higher rate of ACEI/ARB use compared to the non-statin group (39% vs. 10%). There were no significant differences in cancer treatment regimens. Anthracycline chemotherapy was administered to 55% of the statin group and 71% of the non-statin group (*p* = 0.12), with a comparable mean cumulative dose (264 ± 44 mg vs. 255 ± 18 mg, *p* = 0.45). Fig. 1 illustrates longitudinal changes in left ventricular ejection fraction (LVEF) from baseline to 3, 6, 9, and 12 months following initiation of chemotherapy.

Baseline LVEF was similar between groups (68.8 ± 6.3% in the statin group and 67.1 ± 5.1% in the non-statin group). At 3 months, LVEF declined in both groups (63.4 ± 4.6% vs. 63.0 ± 5.2%, respectively). Thereafter, the statin group maintained LVEF (63.3 ± 5.5% at 6 months, 63.6 ± 5.0% at 9 months, and 63.1 ± 5.4% at 12 months), whereas the non-statin group showed progressive decline (61.5 ± 5.7%, 61.1 ± 5.8%, and 60.3 ± 6.1%, respectively). After adjustment for age, hypertension, ACE inhibitor/angiotensin receptor blocker (ACEI/ARB) use, anthracycline exposure, and baseline LVEF in the longitudinal model, the decline in LVEF over time was significantly attenuated in patients receiving statin therapy (statin use-by-time interaction: estimate, 0.27; SE, 0.10; *P* = 0.005). When stratified by ACEI/ARB therapy, no significant difference in LVEF change was observed between users and non-users (−4.2 ± 5.5% in ACEI/ARB and − 4.1 ± 4.9% in non-ACEI/ARB; ACEI/ARB use-by-time interaction: estimate, 0.10; SE, 0.10; *P* = 0.324 after adjustment for age, hypertension, anthracycline exposure, baseline LVEF, and statin use). When Cancer therapy-related cardiac dysfunction (CTRCD) was defined as a decline in LVEF of ≥10 percentage points to an LVEF <53% [9], 23 patients developed CTRCD, including 2 patients who were receiving statin therapy. No patients discontinued trastuzumab therapy because of CTRCD.

## 4 Discussion

Despite having a higher burden of cardiovascular risk factors, the decline in LVEF over time was attenuated in patients receiving statin therapy. A previous study evaluating LVEF during trastuzumab therapy compared baseline LVEF with the final LVEF, defined as the last LVEF measurement closest to the final trastuzumab treatment, and reported that concomitant statin use was associated with a lower risk of cardiotoxicity in women with HER2-positive breast cancer receiving trastuzumab-based therapy with or without anthracyclines [2]. In contrast, a potentially novel observation of the present study was the temporal pattern of LVEF changes during the 12-month follow-up. Although the initial decline in LVEF at 3 months was comparable between patients with and without statin use, the trajectories subsequently diverged, with greater preservation of LVEF among statin users becoming apparent at 9 months and most pronounced at 12 months. These findings suggest that statin use may not prevent the early decline in LVEF associated with trastuzumab therapy but may be associated with attenuation of its subsequent progression. This delayed association may reflect the cumulative pleiotropic effects of statins, including attenuation of oxidative stress and reactive oxygen species, preservation of HER2-mediated cardiomyocyte survival signaling, improvement of endothelial function and nitric oxide bioavailability, and anti-remodeling effects, although the underlying mechanisms remain uncertain [3]. Importantly, potential cardioprotection by statins should be considered alongside oncologic efficacy. In a recent post hoc analysis of 4804 patients with early HER2-positive breast cancer in the APHINITY trial, statin use was not associated with invasive disease-free survival, distant relapse-free interval, or overall survival, providing no clinical signal of adverse oncologic effects during HER2-targeted therapy [10]. However, further studies evaluating both cardiovascular and oncologic outcomes are warranted. This study has several limitations. First, although most baseline statin users (97%) had a history of dyslipidemia and continued statin therapy during follow-up, detailed information on statin type, dose or intensity, and adherence was unavailable. Therefore, the potential influence of these treatment-related factors could not be assessed. Second, although the echocardiographic data were reviewed by physicians independent of the study investigators, the assessment was not performed in a fully blinded manner. Third, longitudinal LVEF data beyond 12 months were not available in the present study. Therefore, the long-term association between statin use and LVEF changes remains uncertain and represents an important direction for future research. Finally, although hypertension was included as a covariate in the adjusted longitudinal model, blood pressure measurements at each echocardiographic assessment and longitudinal changes in antihypertensive therapy were not systematically available. Given the higher prevalence of hypertension among statin users and the load dependence of LVEF, we cannot exclude the possibility that differences in afterload or changes in antihypertensive treatment contributed to the observed LVEF trajectories. Our findings should be interpreted as hypothesis-generating and associative rather than as evidence of a causal cardioprotective effect of statins. Prospective randomized trials are warranted to determine whether statins should be recommended as a cardioprotective strategy during trastuzumab therapy.

## CRediT authorship contribution statement

**Kotaro Nochioka:** Writing – original draft, Visualization, Software, Resources, Project administration, Methodology, Investigation, Formal analysis, Data curation, Conceptualization. **Yosuke Terui:** Writing – review & editing, Project administration, Investigation, Data curation. **Aika Udagawa:** Writing – review & editing, Visualization, Software, Formal analysis. **Shintaro Kato:** Writing – review & editing, Visualization, Validation, Formal analysis. **Shigeru Toyoda:** Writing – review & editing, Investigation, Data curation. **Masaya Shimojima:** Writing – review & editing, Investigation, Data curation. **Yasuhiro Izumiya:** Writing – review & editing, Investigation, Data curation. **Toshiyuki Yano:** Writing – review & editing, Investigation, Data curation. **Takeshi Kitai:** Writing – review & editing, Investigation, Data curation. **Satoshi Yasuda:** Writing – review & editing, Supervision. **Koichiro Sugimura:** Writing – review & editing, Supervision, Resources, Project administration, Methodology, Investigation, Funding acquisition, Data curation, Conceptualization.

## Ethics declaration

Written informed consent to take part in the study and to publish the article has been obtained from all participants or their legal representatives. The privacy rights of participants have been observed.

This study was performed in compliance with relevant laws, regulatory frameworks and guidelines where the research took place.

This study was approved by the The Ethics Committee of Tohoku University Graduate School of Medicine. (Approval No. 2024-1-573)

## Funding

This work was supported by 10.13039/100009619Japan Agency for Medical Research and Development (Tokyo, Japan) grant 18ek0210084h0002 (Dr Sugimura).

## Declaration of competing interest

The authors declare that they have no known competing financial interests or personal relationships that could have influenced the work reported in this paper.
