# Supplementary material for: Association of statin use and left ventricular function in patients with breast cancer receiving trastuzumab
Source: Am Heart J Plus. 2026 Sep 5;70:100871. doi: 10.1016/j.ahjo.2026.100871 (PMC13587717; doi:10.1016/j.ahjo.2026.100871)
Supplement: Supplementary file 1 [file mmc1.docx]

**Supplemental Material**

**Supplemental Figure 1. Study diagram**

**Supplemental Table 1. Comparison of baseline characteristics between patients included in and excluded from the study**

|  | **Overall (N=624)** | **Included patients (n=215)** | **Excluded**  **Patients**  **(n=409)** | **P value** |
| --- | --- | --- | --- | --- |
| Age, mean (SD) | 56.1 (12.1) | 56.6 (11.4) | 55.9 (12.5) | 0.456 |
| Female, n (%) | 624 (100) | 215 (100) | 409 (100) | NA |
| BMI, mean (SD) | 23.2 (4.1) | 22.8 (3.8) | 23.5 (4.3) | 0.052 |
| Cardiovascular risk factors |  |  |  |  |
| Hypertension, n (%) | 156 (25.0) | 54 (25.1) | 102 (24.9) | 1.000 |
| Dyslipidemia, n (%) | 165 (26.4) | 61 (28.4) | 104 (25.4) | 0.486 |
| Diabetes mellitus, n (%) | 57 (9.1) | 17 (7.9) | 40 (9.8) | 0.532 |
| Current or ex-smoker, n (%) | 92 (14.7) | 34 (15.8) | 58 (14.2) | 0.669 |
| History of heart disease, n (%) | 41 (6.6) | 16 (7.4) | 25 (6.1) | 0.646 |
| Medication |  |  |  |  |
| Beta-blocker, n (%) | 19 (3.1) | 7 (3.3) | 12 (2.9) | 1.000 |
| RAS inhibitors, n (%) | 86 (13.8) | 31 (14.4) | 55 (13.5) | 0.850 |
| Statins, n (%) | 101 (16.2) | 31 (14.4) | 70 (17.2) | 0.435 |
| Breast cancer therapy |  |  |  |  |
| Anthracycline, n (%) | 466 (76.3) | 147 (68.4) | 319 (80.6) | 00.001 |
| Trastuzumab, n (%) | 291 (47.6) | 215 (100) | 76 (19.2) | <0.001 |
| Anthracycline and Trastuzumab | 189 (30.9) | 147 (68.4) | 42 (10.6) | <0.001 |

Missing values were excluded from the denominator when calculating percentages.
